# Assessment of Woodcrete Using Destructive and Non-Destructive Test Methods

**DOI:** 10.3390/ma15093066

**Published:** 2022-04-22

**Authors:** Ashraf A.M. Fadiel, Taher Abu-Lebdeh, Florian Ion T. Petrescu

**Affiliations:** 1 Department of Civil Engineering, Omar Al-Mukhtar University, El-Bieda P.O. Box 919, Libya; ashraf.fadiel@omu.edu.ly; 2 Department of Civil, Architectural and Environmental Engineering, North Carolina A and T State University, Greensboro, NC 27411, USA; taher@ncat.edu; 3 “Theory of Mechanisms and Robots” Department, Faculty of Industrial Engineering and Robotics, University Politehnica of Bucharest, Splaiul Independentei Street 313, 060042 Bucharest, Romania

**Keywords:** non-destructive testing, woodcrete, rebound hammer test, ultrasonic pulse velocity test, compressive strength, wood shavings

## Abstract

Utilizing solid wastes and industrial by-products as a partial replacement for raw materials has become an acceptable practice among researchers and scientists in the civil engineering field. Sawdust and wood shavings are not an exception; they are being used in concrete as a partial or total replacement for some of its constituents. The main goal of this research is to establish a relation between destructive and non-destructive testing for concrete containing wood shavings as a partial replacement of sand (woodcrete). With this type of material existing, thus the need to understand the behavior of such material becomes urgent and evokes the need to ease the process of the assessment and the evaluation of such materials and therefore provide more understanding of its behavior. In addition to the conventional concrete mix, five mixes of woodcrete were made by replacing fine aggregate by volume with wood shavings at different replacement levels varied from 5% to 50%. Cubic samples were tested at the age of 90 days using nondestructive tests (NDT), namely, rebound hammer test and ultrasonic pulse velocity test. Then, the specimens were tested using a conventional compressive test using a universal compression testing machine. Statistical analysis was performed to establish empirical relations between destructive and non-destructive results. The dynamic modulus of elasticity was calculated, and some formulas to estimate the (compressive) strength of woodcrete using NDT results were proposed and tested against experimental results and showed acceptable results.

## 1. Introduction

The world is changing faster and faster, with biomimetics and bioengineering penetrating deep into industries of all kinds, including the concrete industry. The acute shortage of raw materials, including sand, has slowly but surely led to the need to scientifically study the replacement of sand concrete with other similar materials that are easier to find in various parts of the world. Various forms of wood and sawdust have recently been tested by scientists, with the aim of using them together with quality cement, instead of sand, to form a resistant concrete, easier to obtain than the classic one formed with sand. The construction sector is considered the largest consumer of raw materials, and concrete is the most used material in the construction field, due to its superiority advantages over other construction materials. Concrete is one of the most essential, widespread, and commonly utilized construction materials due to its benefits over other construction materials, because of the convenience of production and handling, as well as the ease of adopting the shape of the mold, which improves handling at the sites [1,2]. When it comes to construction, two materials are often installed and these are wood and concrete. Engineers and builders have already figured out which material is best for a particular use. However, none of them are considered to be the best in all respects. Wood may be better in one situation, while concrete may be better in another. The wood is organic. There are cells in the wood that make it “alive”. Most forests are brown, ranging from light brown to dark brown, although some forests appear lighter in color because they give it a fleshy white color. Wood is actually an aggregate of cellulose, whose fibers are very compact. This property makes it resistant to external forces and compression. In its state of life, wood is part of the stem or trunk of the tree that is used as a passage for nutrients and water that comes from the roots to the leaves. In addition to construction, wood can be used for other important purposes, such as refilling, packaging, and even papermaking. The wood or trees grow by expanding inside their trunk, and so it grows in diameter and produces more growth rings that are said to be able to tell the age of the tree itself. Wood can also be part of its two major classes. The heartwood is the heart of the tree. It is the inner wood that is considered to be older. Most experts call this part dead wood, but others disagree because it can still be degraded. The sapwood, on the other hand, is the outer part and is considered to be the younger wood. It is primarily responsible for the management of nutrients in the tree. In addition, wood can also be classified as hard or soft. Oakwood is hardwood, while pine wood is soft. Natural sand, gravel, and crushed rock are undeniably important components of concrete and mortars. A great portion of concrete is a mix of fine and coarse aggregates approximately 80% of total concrete volume [1]. Fine aggregate extracted from coastal locations is one of the major sources of sand used in concrete in Libya, particularly in places next to the Mediterranean Sea. The extraction of sand from coastal areas has had a negative impact on the biological life of many marine organisms, necessitating the search for new materials that could be used as substitutes for original materials used in producing concrete without having a significant impact on mechanical properties, durability, and toughness, etc. [2].

The use of industrial wastes as a substitute for some concrete components has emerged as one of the viable approaches for removing the solid wastes and end-used materials from the environment and reducing their negative impact. This kind of practice is essential to reduce the consumption of raw materials associated with the concrete industry. Several studies have been carried out in order to identify alternatives to the aggregates used in concrete. Waste glass, rubber tire scrap, quarry ash, marble dust, wood shavings, sawdust, coal bottom ash, and granulated blast-furnace slag were among the alternatives considered [3,4,5,6,7,8,9,10,11,12]. Earlier studies demonstrated that partially replacing fine aggregates with aggregates generated from solid wastes such as wood and glass industries, marble quarries, and aggregates generated from end-of-life tires yield concrete with acceptable properties, and in some cases even improved some non-mechanical properties such as thermal insulation and sound insulation, as well as an increase in the ability to absorb energy and shock when compared to conventional concrete [12,13,14,15]. The main goal of this research is to utilize non-destructive testing techniques (NDT) to evaluate woodcrete by using destructive and non-destructive testing methods and establishing a correlation between the compressive strength estimated peer BS EN 12390-3:2009 test standard and the rebound number test ASTM C805-08 and pulse velocity test ASTM C597-09. The use of more than one NDT method in comparison with the DT (destructive tests) method would provide a better correlation and would lead to a more reliable strength estimation. This substantially would lead to more understanding of the behavior of woodcrete and increase the awareness of such practice.

Agar-Ozbek et al., have investigated porous wood-concrete with improved strength with testing at different scales [16]. Belytschko and Black have studied the elastic crack growth in finite elements of wood concrete [17]. Brake et al., have studied the flexural strength and fracture size effects of pervious wood concrete [18]. Deppe has analyzed the production and application of cement-bonded wood chipboards [19]. Gunasekaran et al. experimented and have studied reinforced lightweight coconut shell concrete beam behavior under flexure [20]. Hameury and Lundstrôm have contributed to the indoor exposed massive wood to a good indoor climate: in situ measurement campaign [21]. In addition, other types of materials have been tried and tested instead of sand. Kaya and Kar, have made an ample study of the thermal and mechanical properties of concretes with styropor [22]. Kevern et al. studied the effects of macro synthetic fibers on previous concrete properties [23]. Khelifa et al. have studied the finite element analysis of flexural strengthening of timber beams with carbon fiber-reinforced polymers [24]. Koohestani et al. have experimentally investigated the mechanical and microstructural properties of cemented paste backfill containing maple-wood filler [25]. Maple wood is even cheaper than sand or other types of wood, and has superior strength, given that maples are quite easy to grow over a wide geographical area of our planet. A uniform indoor climate, with minor variations in temperature and relative humidity, helps to establish a healthy and comfortable environment for the occupants. It is a well-known fact that the thermal mass of the building envelope counteracts strong temperature changes, (for example, due to solar radiation). However, the fact that there is something like “hydric”, which antagonizes strong variations in humidity, is less common. Here, “wet mass” means the vapor-absorbing capacity of the surrounding surfaces, which is capable of buffering variations in humidity within a space. This would be beneficial in rooms where the generation of moisture (for example, due to human activities) and the extraction of moisture (by ventilation) do not coincide. In this regard, VTT (Espoo, Finland) has conducted numerical investigations [26], which have shown that the wooden lining has a favorable effect on the relative humidity in the bedrooms, a favorable and healthier effect than that of sand or other known materials, which is ventilated, only during the day. In order to validate the interpretation of these calculations and to obtain some practice-oriented quantifications of the moisture buffering effects of different types of inner liners (based on wood or cellulose fiber products) under defined boundary conditions, a series of comparative tests were designed by the Fraunhofer Institute for Building Physics (IBP) in Holzkirchen, Germany [26]. If at one time traditional brick and wood were almost removed from the construction as too classic, and untreated wood too fireproof, today things are starting to change so that wood of any kind becomes an important building material again if processed and used together with other materials and components. Bashar et al. have made some statistical models for concrete containing wood chipping as a partial replacement for the fine aggregate [27]. Naik et al. used CLSM (Controlled Low Strength Materials) containing mixtures of coal ash and new pozzolanic material [28]. Coal was tried instead of wood, coal ash, and wood ash, which were otherwise discarded anyway so that not even the wood was consumed in the mixed materials but only its ash and that of the burned coal. Okino and others have used and studied chipboard glued wood with a mixture of eucalyptus and rubberwood [29]. The properties of fresh and hardened concrete using agricultural waste as a partial replacement for coarse aggregates have also been studied [30]. The use of wood ash in the manufacture of concrete has been resumed since 2012 [31]. We are once again encountering bamboo and wood fibers combined with cement for use in the sustainable regeneration of infrastructure [32]. The design of low-density wood-cement chipboard was designed to finish the interior walls [33]. The study of the potential of wood waste ash as a concrete additive was presented in other papers [34,35,36,37,38,39].

The production of cement used in concrete is a huge source of CO_2_ emissions, so the more we can recycle existing concrete, the better. Here is a new study that shows that discarded concrete becomes even stronger than it was before when wood waste was added. Concrete is made by mixing an aggregate, such as gravel, with water and cement. Once the mixture has hardened, the cement hardens and binds to the aggregate to form a solid block of material. Driven by Yuya Sakai, a scientist at the University of Tokyo, pieces of such concrete were ground into a powder, then added water along with the lignin from the wood waste. Lignin is a highly crosslinked organic polymer and is a key component of supporting tissue in vascularized plants (water conductors)-this is what gives wood its rigidity. The mixture was then heated simultaneously and placed under high pressure. It was found that by precisely adjusting variables such as the concrete/lignin ratio, water content, temperature, plus the amount and duration of pressure, lignin turned into a very effective adhesive, gluing the pieces of concrete powder together. When subsequently tested, it was found that recycled concrete has a higher bending strength than the original concrete from which it was made. As an added bonus, due to its lignin content, the material should probably biodegrade once discarded. In addition, scientists believe that lignin from other plant sources (such as agricultural waste) could be used instead. Finally, it may even be possible to create a new “virgin” concrete, in which lignin is used instead of cement [40].

During the study, an experimental investigation was performed to estimate the compressive strength of wood concrete using NDT test methods using DT results. The dry unit weight was reduced by the addition of wood chips, a reduction of up to 36% to a replacement level of 50%. The reduction in the dry unit weight of wood-concrete is due to the decrease in the weight of the conventional aggregate, which is replaced with lighter material (wood chips) and the increase in air gaps as the amount of shaved wood has increased. Water absorption has increased with the increase in the number of wood chips; however, up to a replacement level of 30%, wood concrete has maintained a water absorption of less than 10%, which is considered a good quality concrete. Compressive strength decreased as the number of wood chips increased; however, concrete mixtures with 5, 10 and 15% wood chips showed a compressive strength of 34.6, 27.6, and, respectively, 20.1 MPa. A formula for estimating compressive strength based on the amount of wood chips was also proposed.

## 2. Materials and Methods

### 2.1. Materials Utilized

Cement:

Portland cement type I (42.5N) complied with ASTM C150-12 was used in this research. The chemical–physical and mechanical properties of the cement are shown in Table 1 and Table 2.

**Figure 1:**
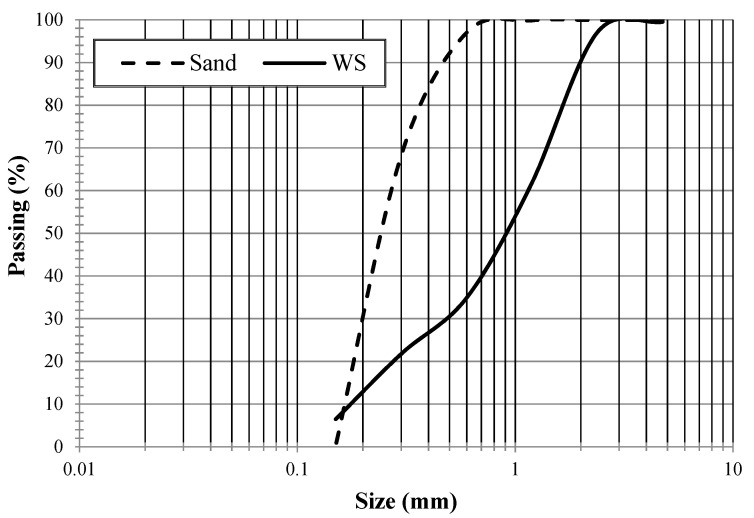
Gradation of sand and wood shavings.

Sand and wood shavings:

The gradation of sand used in this study conformed to the ASTM C33 standards. The particles distribution for sand and wood-shaving is shown in (Figure 1). The wood shavings (WS) were used in saturated surface dry conditions throughout the course of the study. It should be mentioned that wood shavings were not exposed to any chemical treatment. Previous studies concluded that wood shavings in saturated surface dry conditions would scatter better in the dry mixture and do not absorb the free water that is intended to hydrate the cement and enhance concrete workability [11,41].

**Figure 2:**
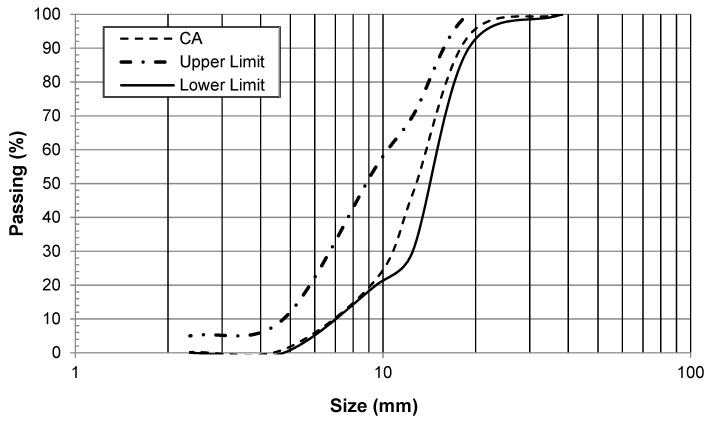
Gradation of coarse aggregate.

Coarse Aggregate: The gradation of coarse aggregate used in this research conformed to the ASTM C33 standards (Figure 2). The physical and mechanical properties of wood shavings, fine, and coarse aggregates are summarized in (Table 3).

### 2.2. Methodology

In order to examine the behavior of woodcrete under non-destructive tests and obtain reliable results using NDT, the specimens were tested using two types of NDT, namely, ultrasonic pulse velocity and rebound hammer tests, and then tested using the compressive test at age of 90 days. Woodcrete mixes were prepared by partial replacement of sand with wood shavings by volume. Five levels of replacement were used, namely, 5%, 10%, 15%, 30%, and 50% in addition to controlling the mix. A total of 18 cubes were prepared, cast, and cured according to ASTM C192.

These mixes were validated by a previous study conducted by [11]. At age of 28 days, the mixes showed acceptable compressive strength up to a 15% level of replacement. The compressive strength was 32, 25.5, and 15.5 MPa for concrete mixes containing 5%, 10%, and 15% of wood shavings, respectively. This was a motivation to conduct further investigation and obtain more insight into the properties of such material, which became a promising practice of utilizing waste wood related to wood industries.

At age of 90 days, samples were taken out of the curing tank and allowed to dry for 24 h. After that, the samples were weighted to calculate the density ASTM C 642-13. The specimen was first tested for the UPV test, then the rebound hammer test, and finally, tested using a compression testing machine as a peer (BS EN 12390-3:2009). The data of each test was recorded and the results were compared and used to derive simple correlations between different test methods.

### 2.3. Test Procedures

#### 2.3.1. Ultrasonic Pulse Velocity Test (UPV)

**Figure 3:**
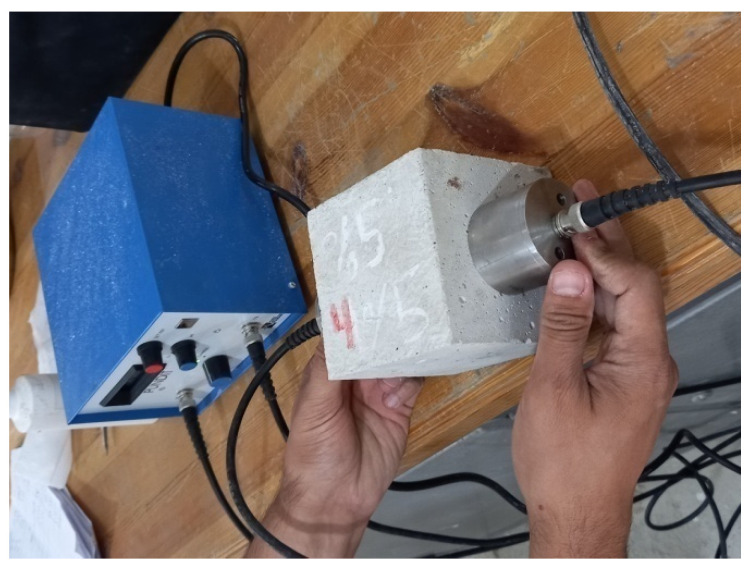
Ultrasonic pulse velocity test setup.

The test is described in ASTM C 597–09 [42], the direct measurement procedure of the UPV test is based on measuring the time of longitudinal stress wave pluses through concrete between two transducers attached to the concrete on the opposite surface of the concrete specimen (Figure 3). Once the distance between the two transducers is measured and the time of transmitting stress waves from one transducer to the second one is obtained the pulse velocity is simply calculated by the following formula:

(1) $$V=\frac{L}{T}$$

where:

*V*: Pulse velocity (m/s), 

*L*: Pulse velocity(m), 

*T*: Time measured in (sec).

In addition to measuring pulse velocity, ultrasound measurements provide a simple non-destructive, and inexpensive technique to estimate the elastic modulus of concrete. The following formula is used to estimate the dynamic modulus of elasticity:

(2) $$V=\sqrt{\frac{E\left(1-\mu \right)}{\rho \left(1+\mu \right)\left(1-2\mu \right)}}$$

where:

*V*: Pulse velocity (m/s);

μ*:* dynamic Poisson’s ratio;

E*:* dynamic modulus of elasticity (MPa);

ρ*:* density (Kg/m^3^).

The are many factors contributing to the variability of the results obtained from the ultrasonic pulse velocity method; such, as cement type, w/c ratio, aggregate size and type, admixtures, age of concrete, positioning and distance between transducers, etc., by careful consideration to aforementioned factors, ultrasonic pulse velocity methods are excellent, simple and inexpensive means for investigating the uniformity and durability of concrete [43,44,45,46].

#### 2.3.2. Rebound Hammer Test

**Figure 4:**
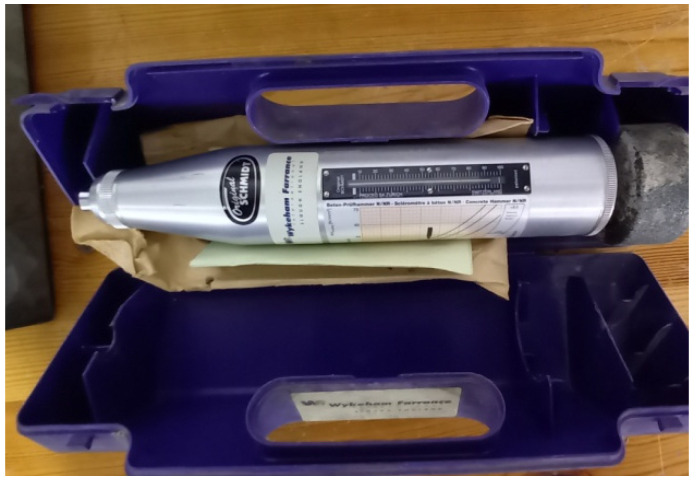
Schmidt Hammer apparatus.

**Figure 5:**
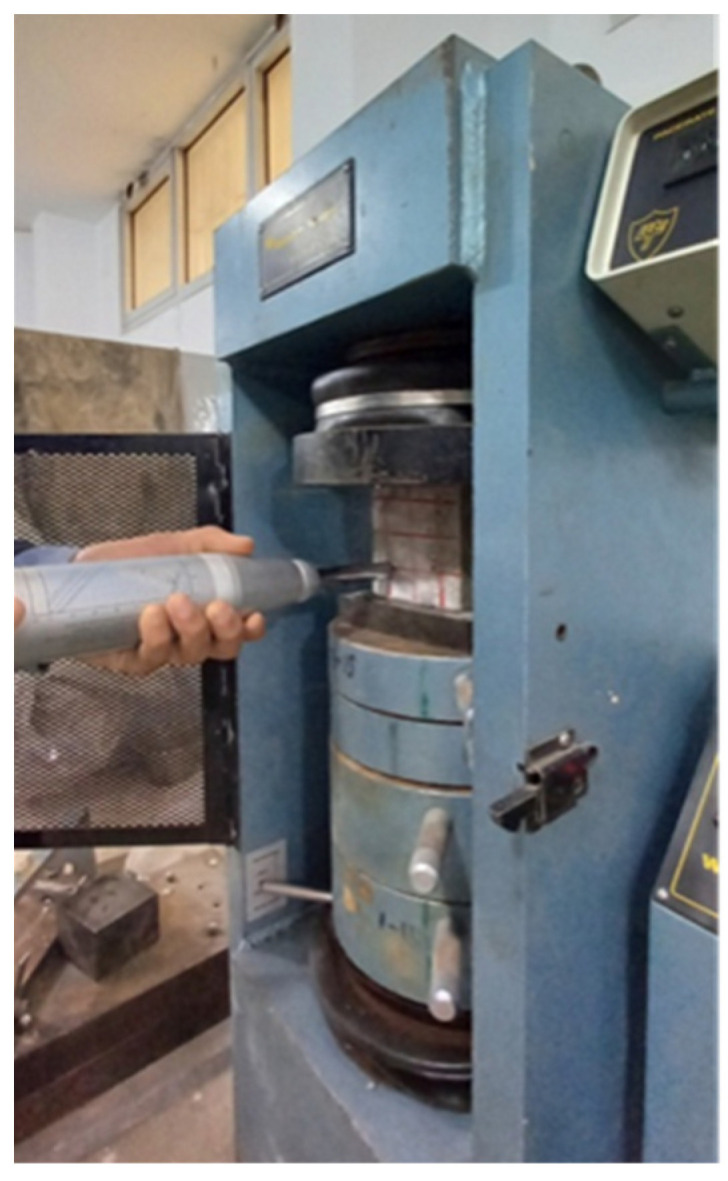
Rebound hammer test setup.

The hammer rebound test is described in ASTM C: 805 [47]. The test is performed by the main Schmidt hammer apparatus (Type N) as shown in (Figure 4). The depth of the tested area shall be at least 100 mm and 150 mm in diameter. The rebound hammer test is based on the principle that the rebound of an elastic mass depends on the hardness of the surface against the mass impinges. First, two opposite faces of the specimen were prepared with abrasive stone to ensure the ground and smooth surface of the specimen, then the measuring points were prepared and located. Later, each specimen was rigidly supported by applying a slight load using a compression testing machine (Figure 5). The hammer should be firmly held in a perpendicular direction to the prepared test surface. At least ten readings were obtained on each face of the specimen. The rebound hammer was kept horizontal in all measurements. The readings were evaluated and recorded.

#### 2.3.3. Compressive Test

The compressive test was performed using a universal testing machine and following the procedure stated in BS EN 12390-3:2009. After the non-destructive testing has been fully completed, the specimens were placed on a compression testing machine and loaded to failure.

## 3. Results and Discussion

### 3.1. The Effect of Wood Shavings on Dry Unit Weight

**Figure 6:**
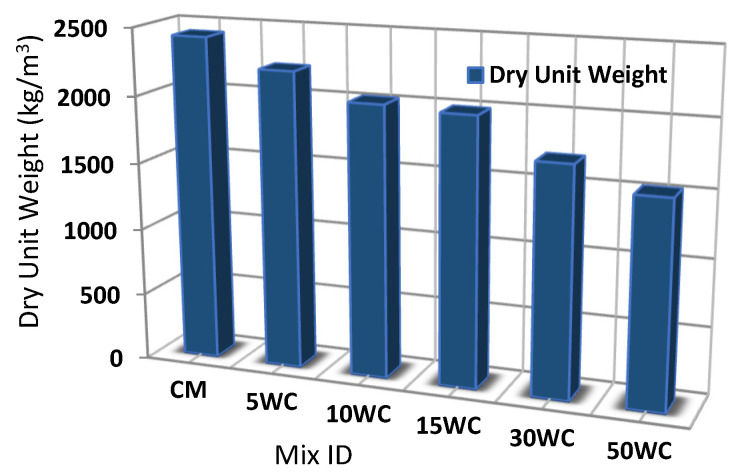
Values of dry unit weight.

The results of the dry unit weight at the age of 90 days were measured according to ASTM C 642-13 and illustrated in Figure 6. As the replacement ratio of wood shaving increased, the dry unit weight decreased. The dry unit weight of the control mix was 2426.7 kg/m^3^ and for woodcrete ranged from 1530 to 2217.4 kg/m^3^. Concrete mixes 5WC, 10WC, 15WC, 30WC, and 50WC showed 8.6%, 16.6%, 17.8%, 29.7%, and 36.9%, respectively, which is a lower dry unit weight compared to the control mix. The reduction in the dry unit weight is due to the lighter weight of wood shavings compared to natural sand and also because of entrapped air content developed in mixes containing wood shavings, which were found to be increased as the amount of the wood shavings increased. The decrease in the dry unit weight is an indication of the reduction of dead load. At 50% replacement level approximately 37% of the dead load was reduced. Mixes with 30 and 50% wood shavings content have a dry unit weight of 1706 and 1530 kg/m^3^, respectively, which is less than1800 kg/m^3^. Therefore, lightweight concrete could be considered a peer of ACI 213R-87.

### 3.2. The Effect of Wood Shavings on Absorption

**Figure 7:**
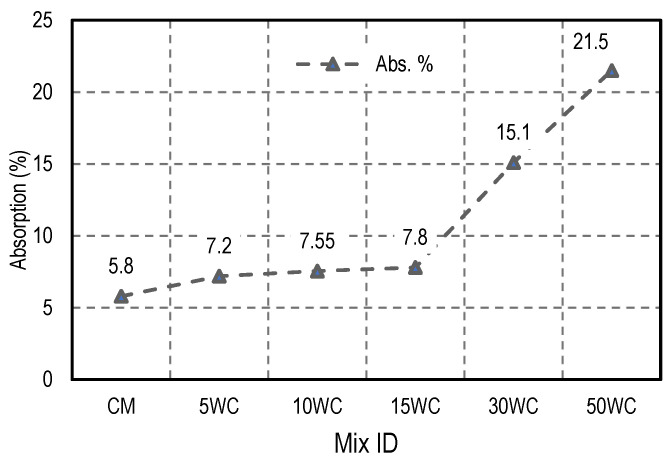
Water absorption values for different mixes.

Water absorption of different mixes was measured based on ASTM C642-13. Water absorption is simply calculated by measuring the increase in mass as a percentage of dry mass the results were presented in (Figure 7). It was observed that the water absorption increased as the amount of wood shavings increased. Water absorption of concrete mixes 5WC, 10WC, 15WC, 30WC, and 50WC increased by 24.1%, 30.2%, 34.5%, 160.3%, and 270.7%, respectively, compared to the control mix. The water absorption of mixes containing up to 15% of wood shavings was less than 10%, which is considered a good quality concrete [48,49]. However, the water absorption sharply increased in woodcrete containing 30% or more of wood shavings. This is obviously due to the nature of wood, which is the ability to absorb water, and for higher wood shavings inclusion the increase can be attributed to the existence of some wood shavings on the surface of the samples which directly contacted water.

### 3.3. The Effect of Wood Shavings on Ultra Pulse Velocity

**Figure 8:**
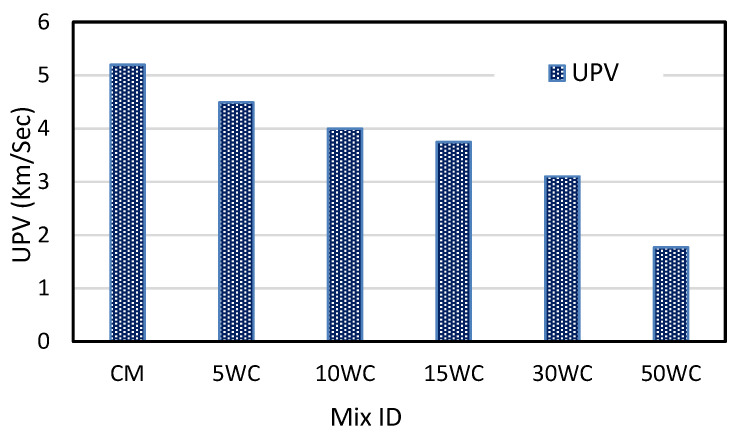
Ultrasonic pulse velocity results.

As the amount of wood shavings increases, the UPV decreases, and the UPV of woodcrete ranges from 1.77–4.49 km/s. The control mix recorded 5.2 km/s and the highest value of UPV was recorded for 5WC, which was 13.7% lower than the control mix. The lowest value of UPV was recorded for the 50WC mix and it was 66% less than CM. Figure 8 represents the UPV values. The reduction in UPV can be attributed to a decrease in solid particles and an increase in air voids as the amount of wood shavings increased, similar conclusions were derived by [43]. 

### 3.4. The Effect of Wood Shavings on Rebound Number

**Figure 9:**
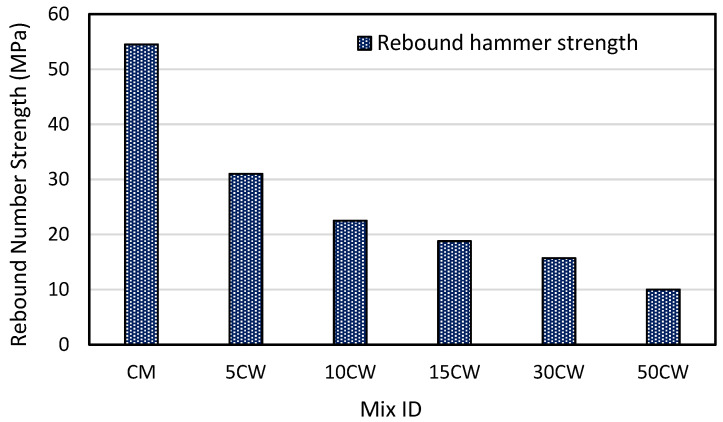
Rebound hammer strength for different mixes.

After conducting the rebound hammer test on all samples, the average of the rebound number is calculated and revised by discarding the readings that differ by six units from the average (ASTM C: 805) [47]. The rebound number decreased as the amount of wood shavings increased, the rebound number decreased by 30.9%, 38.4%, 47.2%, 51.4%, and 63.2% for 5WC, 10WC, 15WC, 30WC, and 50WC mixes, respectively, compared to the control mix which had an average rebound number of 49.2. Figure 9 represents the rebound number strength for different mixes. The rebound numbers used to estimate the compressive strength of the control mix and the mixes contained wood shavings and were found to be 54.5, 31, 22.5, 18.8, 15.7, and 10 MPa for CM, 5WC, 10WC, 15WC, 30WC, and 50WC, respectively. The rebound hammer test results affect the surface of concrete and the existence of voids and aggregates [48,49], and the presence of voids would yield a lower rebound number. In addition, the wood shavings particles are less stiff than normal aggregate; hence, they absorb more energy, which results in a lower rebound number that is noticeable at the higher wood shavings amount (30–50%).

### 3.5. The Effect of Wood Shavings on Compressive Strength

**Figure 10:**
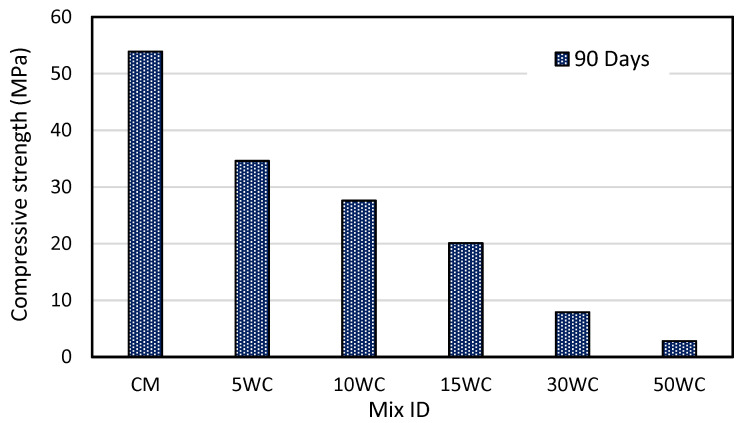
Destructive compressive strength at 90 days.

The test was conducted according to (BS EN 12390-3:2009) [50]. At the age of 90 days, the specimens were tested and the average of three specimens was recorded. Figure 10 illustrates the compressive strength of different concrete mixtures containing wood shavings at 90 days. Concrete mixes 5WC, 10WC, 15WC, 30WC, and 50WC showed lower compressive strength than the control mix, at 35.8%, 48.8%, 62.7%, 85.3%, and 94.8%, respectively, lower compressive strength compared to the control mix which has a compressive strength of 53.9 MPa. Though 5WC, 10WC, and 15WC mixes have a compressive strength of 34.6, 27.6, and 20.1 Mpa, respectively. The regression analysis of data reveals that compressive strength correlated in an exponential custom, as the amount of wood shavings increased (Equation (3)) and the coefficient of regression (R^2^) was found to be 0.998, the percentage difference between actual values of compressive strength and the predicted strength based on (Equation (3)) ranged from ±(3–6%).

(3) $$f_{c}=48.373\;e^{-0.061\;w}$$

where:

$f_{c}$: Predicted compressive strength (MPa),

w: Amount of wood shavings (%).

The decrease in compressive strength as stated in [11] can be attributed to the lack of bond between wood shavings particles and the surrounding cement paste, which leads to the weakening of the interfacial transition zones (ITZ) especially surrounding WS particles. Wood shavings particles have stiffness less than sand, and the water released from wood shavings particles during the mixing procedure increases the free water hence increasing the actual w/c ratio. As shown above, up to a 15% replacement level, the mixes showed an acceptable compressive strength which makes them suitable for partition walls, nonstructural elements, and concrete bricks production. Table 4 summarizes the average of three tests of destructive and non-destructive tests along with the dry unit weight values for different mixes. The confidence intervals were added to the results to explain the range of the results.

### 3.6. The Effect of WS on the Dynamic Modulus of Elasticity 

**Figure 11:**
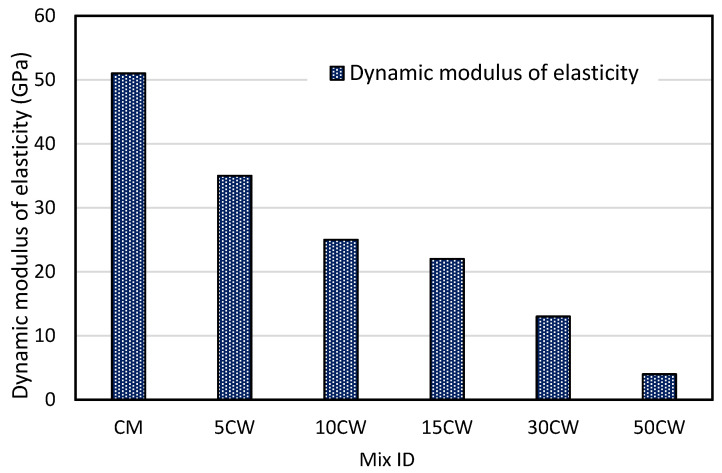
Values of dynamic modulus of elasticity.

It was mentioned earlier that there is a unique relation between UPV and the dynamic modulus of elasticity which is governed by Equation (2) mentioned above. By plugging the values of pulse velocity (*V*) and dry unit weight (ρ), it is assumed that the value of dynamic poisons ratio (*μ*) is equal to 0.28 [51,52]. Figure 11 shows the values of dynamic modulus of elasticity for different percentages of wood shavings [53,54].

The values of dynamic modulus of elasticity were found to be decreased as the amount of wood shavings increased and ranged from 35 to 4 GPa for wood crate mixes, the control mix recorded 51 GPa, and the lowest value recorded by the 50CW mix was 4 GPa. The value of the dynamic modulus of elasticity is affected directly by the values of dry unit weight and pulse velocity, since those values decreased as the amount of wood shavings increased, therefore the dynamic modulus of elasticity decreased.

### 3.7. Relation between Compressive Strength and UPV

The results obtained by the ultrasonic velocity test were used to derive an equation to predict the compressive strength of woodcrete. The regression analysis was performed and yielded that the compressive strength and UPV correlated in an exponential way, as the amount of wood shavings increased (Equation (4)) and the R-squared (R^2^) were found to be 0.979.

(4) $$f_{c}=0.4693\;e^{0.979\;V}\;$$

where:

$f_{c}$: Predicted compressive strength (MPa);

V: Pulse velocity (km/sec).

**Figure 12:**
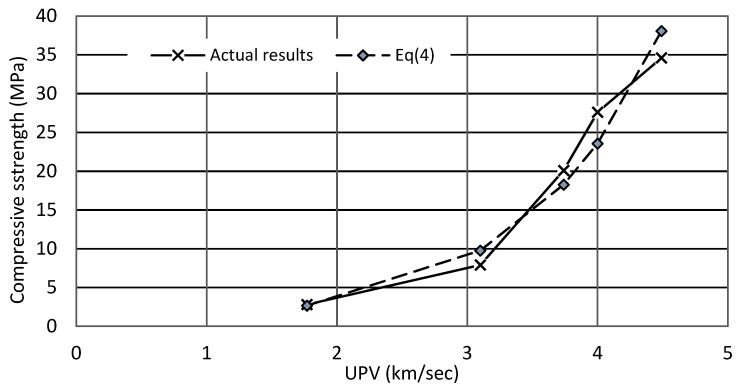
Actual compressive and predicted compressive based on Equation (4).

The percentage difference between actual values of compressive strength and the predicted strength based on (Equation (4)) ranged from ±(5.2–14.6%) for mixes containing 5–15% and 50% wood shavings. A higher difference (33.4%) was observed for the mix containing 30% of wood shavings, Figure 12. The relation between actual compressive strength and predicted compressive strength is based on (Equation (4)).

### 3.8. Relation between Compressive Strength and Rebound Hammer Strength

The results obtained from the rebound hammer test were used to derive an equation to predict the compressive strength of woodcrete. The regression analysis was performed and showed that the compressive strength (DT) and rebound hammer compressive strength correlated in logarithmic mode, as the amount of wood shavings increased (Equation (5)) and the coefficient of regression (R^2^) was found to be 0.93.

(5) $$f_{c}=30.315\;ln\left(Rc\right)-69$$

where:

$f_{c}$: Predicted compressive strength (MPa);

Rc: Rebound hammer compressive strength (MPa).

**Figure 13:**
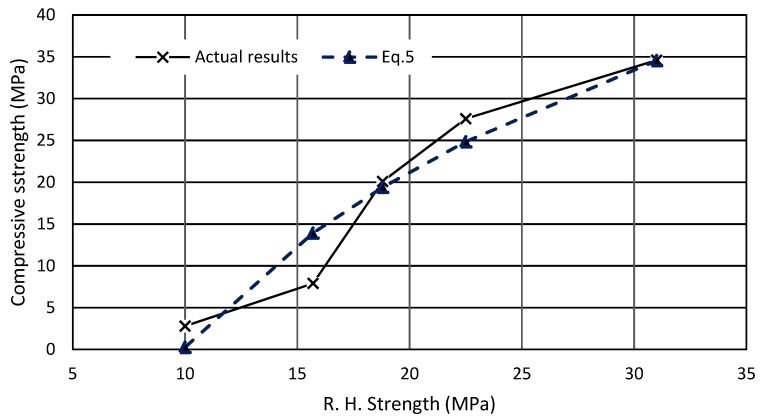
Actual compressive and predicted compressive based on Equation (5).

The percentage difference between actual values of compressive strength and the predicted strength based on (Equation (5)) were (3–10%) less than actual values for mixes containing 5–15% wood shavings. For mixes containing 30–50% of wood shavings, the equation yielded a higher percentage difference of approximately ±90%, Figure 13. The relation between actual compressive strength and predicted compressive strength is based on (Equation (5)).

### 3.9. Combined Methods Analysis

The use of one NDT method to estimate concrete strength would not be adequate. Therefore, the need to combine the results obtained from different methods would yield more reliable and judicious results. The need to use such an approach became handy since the results obtained by Equation (5) had some discrepancies, especially at higher wood shavings content which was around 90%. The multiple regressions were carried out to estimate the compressive strength using results obtained from the rebound hammer test and ultrasonic velocity test. The results obtained from the rebound hammer test were used to derive an equation to predict the compressive strength of woodcrete. The regression analysis yielded Equation (6) and the coefficient of regression (R^2^) was found to be 0.94.

(6) $$f_{c}=-17.2478+4.0631\;V+1.1197\;Rc$$

where:

$f_{c}$*:* Predicted compressive strength (MPa);

V: Pulse velocity (Km/sec);

Rc: Rebound hammer compressive strength (MPa).

The percentage difference between actual values of compressive strength and the estimated compressive strength by Equation (6) were −3.2, 12.3, 5.4, −63.6, 59.2% for 5WC, 10WC, 15WC, 30WC, and 50WC, respectively. The negative sign indicates that (Equation (6)) overestimated the value of compressive strength. From (Equation (6)), the compressive strength is estimated based on the pulse velocity, as well as the compressive strength estimated by the rebound hammer, and since these values decrease with the increase in the amount of wood shavings, logically, the values estimated by this equation for mixtures containing high percentages of wood shavings would have some discrepancy. In all cases, the equation can be considered valid for estimating the compressive strength of mixtures containing wood shavings percentages less than 15%.

## 4. Conclusions

An experimental investigation was conducted to estimate the compressive strength of woodcrete using NDT test methods with aid of DT results. Conclusions are drawn as follows.

The dry unit weight was reduced by adding wood shavings, and up to 36% reduction was recorded at a 50% level of replacement. The reduction of the dry unit weight of woodcrete is due to the reduction of the weight of conventional aggregate that is replaced with lighter material (wood shavings) and the increase in air voids as the amount of wood shaving was increased. 

The water absorption increased as the amount of wood shavings increased; however, up to a 30% level of replacement, the woodcrete maintained water absorption of less than 10%, which is considered a good quality concrete.

The compressive strength declined as the amount of wood shavings increased, nevertheless, woodcrete mixes with 5%, 10%, and 15% wood shavings recorded compressive strength of 34.6, 27.6, and 20.1 Mpa, respectively. In addition, a formula to estimate compressive strength based on wood shavings amount was proposed (Equation (3)). The formula predicted the compressive strength with a small margin of error varied from ±(3–6%).

The ultrasonic pulse velocity test results were used to establish a correlation with destructive test results. The generated equation (Equation (4)) estimated the compressive strength with an error percentage up to 33.4%.

Up to 15% wood shavings content, the use of rebound hammer test provided reliable results in comparison to DT results. The maximum difference percentage was 18%. On the other hand, mixes that contained more than 30% of wood shavings had rebound hammer strength with a percentage difference exceeding 115%. 

Correlation between the compressive strength values by destructive test and rebound hammer test was completed. The resulting formula (Equation (5)), reduced the percentage difference to 10% for mixes containing up to 15% wood shavings, and the percentage difference reduced to less than 90% for mixes containing 30% and more wood shavings. 

The correlation of combined NDT tests results with DT results for woodcrete was conducted and the resulting formula showed a good correlation (R^2^ = 0.94). The estimated compressive strength of mixes contained up to 15% of wood shavings differed only by a maximum of 12%. At higher levels of replacement, the percentage difference reached 63.6%.

The effect of wood shavings on the dynamic modulus of elasticity was considered with the aid of UPV results. The dynamic modulus of elasticity of woodcrete varied from 4–35 GPa. The dynamic modulus of elasticity is an indication of concrete quality and is used to evaluate concrete when exposed to severe conditions.

## Figures and Tables

**Table 1:** Physical and mechanical properties of Portland cement.

Property	Value	Standard
Normal consistency (%)	27.5	---
Initial setting time, (min)	115	>45
Final setting time, (min)	220	<375
Soundness, (mm)	1	<10
Fineness (%)	94.2	
Specific Gravity	3.15	
Compressive strength, (MPa)	3 days = 17	12 MPa
	7 days = 30.5	19 MPa

**Table 2:** Chemical properties of Portland cement.

Oxide Composition	Weight (%)
CaO	63.56
SiO_2_	20.29
Al_2_O_3_	5.65
Fe_2_O_3_	3.3
MgO	2.06
SO_3_	2.7
Na_2_O	0.19
K_2_O	0.85
Cl	0.007
TiO_2_	0.34
MnO	0.043

**Table 3:** Properties of fine, coarse aggregates and wood shavings.

Property	Fine Aggregate	Wood Shavings	Coarse Aggregate
Specific gravity	2.67	1.18	2.59
Absorption (%)	0.3	19	1.4
Bulk density (kg/m^3^)	1767	465	1534
Impact value (%)	---	---	16.4
Crushing Value	---	---	24

**Table 4:** The results of destructive and non-destructive tests for different mixes.

Mix ID	Dry Unit Weight	UPV	Rebound Hammer Strength	Compressive Strength
	Kg/m^3^	Km/Sec	(MPa)	(MPa)
CM	2426.7 ± 9.9	5.2 ± 0.18	54.5 ± 0.90	53.9 ± 0.25
5WC	2217.4 ± 5.13	4.49 ± 0.13	31 ± 0.90	34.6 ± 0.90
10WC	2022.7 ± 3.02	4 ± 0.23	22.5 ± 0.66	27.6 ± 0.75
15WC	1994.7 ± 3.28	3.75 ± 0.07	18.8 ± 0.50	20.1 ± 1.08
30WC	1706 ± 1.55	3.1 ± 0.35	15.7 ± 0.45	7.9 ± 0.66
50WC	1530.4 ± 6.07	1.77 ± 0.09	10 ± 0.50	2.8 ± 0.16

## Data Availability

Not applicable.
